# The anti-inflammatory effect of a magnoliae cortex and Zea mays L. extract mixture in a canine model of ligature-induced periodontitis

**DOI:** 10.1186/s12917-024-04243-0

**Published:** 2024-09-28

**Authors:** Se Eun Kim, Sun Young Hwang, Yong Ho Park, William C. Davis, Kun Taek Park

**Affiliations:** 1 Small Animal Clinical Research Institute, Haemaru Referral Animal Hospital, Seongnam, 13590 Korea; 2 Department of Veterinary Clinical Science, College of Veterinary Medicine, Seoul National University, Seoul, 08826 Korea; 3 Department of Veterinary Microbiology, College of Veterinary Medicine, Seoul National University, Seoul, 08826 Korea; 4 Department of Veterinary Microbiology and Pathology, Washington State University, Pullman, WA 99164 USA; 5 Department of Biotechnology, Inje University, Gimhae, 50834 Korea

**Keywords:** Periodontitis, Tumor necrosis factor-α, Magnoliae cortex extract, *Zea mays* L. extract, Dog

## Abstract

**Background:**

Periodontitis is common in dogs. It is characterized by destruction of the supporting tissues of the teeth due to the host-immune response triggered by plaque. Magnoliae cortex and Zea mays L. extract showed anti-inflammatory and anti-microbial effects, respectively. This study aimed to evaluate improvement in periodontitis following the administration of Magnoliae cortex and *Zea mays* L. extract in dogs. Periodontitis was experimentally induced in 10 beagle dogs. Five dogs were administered 40 mg of Magnoliae cortex extract and 20 mg of *Zea mays* L. extract orally once per day for 2 months (MZ group), whereas the other group received empty gelatin capsules (control group). Periodontal clinical parameters, complete blood count, serum chemistry parameters, and tissue inflammatory cytokines and chemokine expression were assessed before and after combined oral extracts administration.

**Results:**

The complete blood count and serum chemistry results of all dogs were within normal ranges. Gingival inflammation in MZ group was significantly better than that in the control group at 4 and 8 weeks post-medication (PM; *p* < 0.05). The periodontal pocket depth and clinical attachment loss at 8 weeks PM in the MZ group were significantly lower than the baseline values (*p* < 0.05). The incidence of bleeding on probing in the MZ group was significantly lower than that in the control group at 4 weeks PM (*p* < 0.05). Throughout the medication period, the percentages of CD4 + and CD8 + T cells were higher and lower, respectively, in the MZ group. However, these differences were only significant at 8 weeks PM. The expression of the inflammatory cytokines IL-1β, IL-6, IL-17, and TNF-α and the chemokine IL-8 in the inflamed tissues was lower in the MZ group, and the two groups showed a significant difference in TNF-α expression.

**Conclusions:**

Combined administration of Magnoliae cortex and *Zea mays* L. extract improved the clinical symptoms of periodontal disease in dogs. This beneficial effect may be partly due to the inhibitory effects of these extracts on the inflammatory response.

**Supplementary Information:**

The online version contains supplementary material available at 10.1186/s12917-024-04243-0.

## Background

Periodontal disease is triggered by biofilms attached to the tooth surface and progresses through inflammatory processes triggered to protect against the pathogenic microorganisms. Mechanical plaque removal is the most effective method for preventing the progression of periodontitis [1]. Antimicrobials can also alleviate periodontal disease by reducing subgingival microbiota; however, long-term antibiotic administration can lead to antimicrobial resistance [2]. Therefore, several canine studies have used plant extracts for the treatment of periodontitis, and some of these extracts have been shown to be effective [3–5].

Magnoliae cortex extract has been used as a remedy for acute diarrhea, vomiting, regurgitation, and dyspepsia. Magnolol and honokiol are the effective compounds derived from Magnoliae cortex. Magnolol suppresses interleukin (IL)-6-induced signal transduction and activates transcription-3, and honokiol has been shown to inhibit the production of tumor necrosis factor (TNF)-α, prostaglandin E2, and nitric oxide expression in vitro [6–8]. *Zea mays* L. extract improves periodontal tissue health, and its combination with Magnoliae cortex extract has shown effective antimicrobial activity against periodontal pathogens in humans [9]. A more recent in vitro study revealed that Magnoliae cortex and *Zea mays* L. extracts modulated multiple inflammatory reactions induced by *Porphyromonas gingivalis* through the inhibition of nitric oxide, prostaglandin E2, extracellular signal-related kinase 1/2, inducible nitric oxide synthase, IL-1β, IL-6, and nuclear factor-κB levels [10].

In a previous canine study, oral administration of a combination of Magnoliae cortex and *Zea mays* L. extracts improved the periodontal status [11]. In that study, the extract combination was administered to dogs for 8 weeks at doses equal to or twice as high as those administered to adult humans. Although the results showed improvement in clinical periodontal indicators, the in vivo anti-inflammatory effects and safety after long-term administration in dogs were not evaluated. Therefore, this study aimed to evaluate the improvement in clinical parameters and changes in the inflammatory state of tissues after long-term administration of a mixture of Magnoliae cortex and *Zea mays* L. extracts at a lower dose in proportion to the body weight of dogs.

## Results

### General status, CBC, and serum chemistry

During the experiment, none of the dogs had an abnormal general health status (i.e., body weight, body temperature, vitality, and respiratory rate). Tables 1 and 2 show the maximum and minimum values for each parameter in the CBC and serum chemistry evaluations during the experimental period, respectively. The CBC and serum chemistry parameter values of all dogs were within the normal ranges throughout the experimental phase, except for a slight decrease in the potassium level prior to periodontitis induction in one dog.

**Table 1:** Ranges of complete blood counts for all dogs during the experimental period

	Measured range		Reference range
	Minimum	Maximum	
WBC (x 10^3^ cells/ml)	6.01	16.2	5.03–17.1
RBC (x 10^6^ cells/ml)	5.5	7.8	5.5–8.68
Hemoglobin (g/dl)	12.3	17.4	12.3–18.0
Hematocrit (%)	37.2	50.3	37.0–55.2
Platelets (x 10^3^ cells/ml)	159	494	143–506
Neutrophils (x 10^3^ cells/ml)	3.49	11.72	3.0–11.8
Lymphocytes (x 10^3^ cells/ml)	1.34	4.58	1.2–4.8
Monocytes (x 10^3^ cells/ml)	0.24	1.49	0.1–2.1
Eosinophils (x 10^3^ cells/ml)	0.15	1.24	0.1–1.3
Basophils (x 10^3^ cells/ml)	0.01	0.41	0–0.5

WBC, white blood cell; RBC, red blood cell.

**Table 2:** Ranges of serum chemistry results for all dogs during the experimental period

		Measured ranges		Reference range
		Minimum	Maximum	
Albumin (g/dl)		2.30	3.27	2.3–3.9
ALP (U/L)		27.60	151.50	20–155
ALT (U/L)		5.20	49.90	3–50
Amylase (U/L)		421.50	985.60	388–1007
AST (U/L)		10.30	28.55	10–37
Total bilirubin (mg/dl)		0.10	0.70	0.1–0.7
BUN (mg/dl)		5.86	17.89	5.0–30.0
Calcium (mg/dl)		9.10	10.61	9.1–11.7
Creatinine (mg/dl)		0.51	1.11	0.5–1.5
Glucose (mg/dl)		70.75	121.13	67–147
Total protein (g/dl)		5.26	7.20	4.9–7.2
Na (mmol/L)		140.00	150.00	139–151
K (mmol/L)		3.40^*^	5.20	3.5–5.2
Cl (mmol/L)		107.00	117.00	104–118

^*^ Out of the reference range in one dog prior to periodontitis induction. ALP, alkaline phosphatase; ALT, alanine transaminase; AST, aspartate transferase; BUN, blood urea nitrogen.

### Clinical parameters of periodontal status

The mean ± standard error values of periodontal indices at 0, 1, 4 and 8 weeks PM are presented with gum tissue images in Table 3 and a supplementary figure (Fig. S1). The baseline values at 0 weeks PM were not significantly different for any of the periodontal parameters. PI mildly increased at 1 week PM in both groups, and a significant difference was observed at 8 weeks PM in the MZ group and at 4 and 8 weeks PM in the control group (*p* < 0.05). The GI decreased significantly at 1 week PM in comparison with the baseline in both groups. Subsequently, the GI gradually decreased in the MZ group, whereas no significant variations were observed in the control group (*p* < 0.05). The PPD results showed a significant decrease in comparison with the baseline at 8 weeks PM in the MZ group (*p* < 0.05), whereas no differences were observed in the control group. The CAL in the MZ group gradually improved at every examination time point (*p* < 0.05); however, the control group showed no significant difference in comparison with the baseline during the medication period. The BoP values in the MZ group at 4 and 8 weeks PM were significantly lower than the baseline values (*p* < 0.05); in contrast, the control group showed no difference throughout the examination period.

**Table 3:** Changes in each periodontal index over the 8 week treatment period

Groups	Parameters	Week 0	Week 1	Week 4	Week 8
	PI	2.48 ± 0.08	2.49 ± 0.07	2.63 ± 0.05	2.68 ± 0.06^†^
	GI	2.43 ± 0.05	1.86 ± 0.07^*^	1.64 ± 0.07^*,†^	1.61 ± 0.07^*,†^
MZ group	PPD	2.13 ± 0.09	2.09 ± 0.09	2.03 ± 0.07	1.86 ± 0.08^*,†,‡^
	CAL	2.40 ± 0.08	2.01 ± 0.09 ^*^	1.81 ± 0.09^*,†^	1.39 ± 0.10^*,†,‡^
	BoP	0.81 ± 0.04	0.69 ± 0.05	0.63 ± 0.05^*^	0.63 ± 0.05^*^
	PI	2.42 ± 0.08	2.44 ± 0.08	2.64 ± 0.05^*^	2.70 ± 0.05^*,†^
	GI	2.43 ± 0.05	1.94 ± 0.06^*^	1.84 ± 0.06^*^	1.84 ± 0.07^*^
Control group	PPD	2.11 ± 0.08	2.14 ± 0.10	2.13 ± 0.09	2.13 ± 0.10
	CAL	2.38 ± 0.08	2.10 ± 0.10^*^	1.90 ± 0.09^*^	1.82 ± 0.11^*,†^
	BoP	0.81 ± 0.04	0.74 ± 0.05	0.78 ± 0.04	0.69 ± 0.05

^*^Significantly different compared to baseline (0 wk, *p* < 0.05), ^†^Significantly different compared to 1 wk (*p* < 0.05), ^‡^Significantly different compared to 4 wks (*p* < 0.05). The MZ group received Magnolia cortex extract 40 mg with Zea mays L. extract 20 mg and the control group received the vehicle only. PI, plaque index; GI, gingival index; PPD, periodontal pocket depth; CAL, clinical attachment loss; BoP, bleeding on probing.

**Fig. 1:**
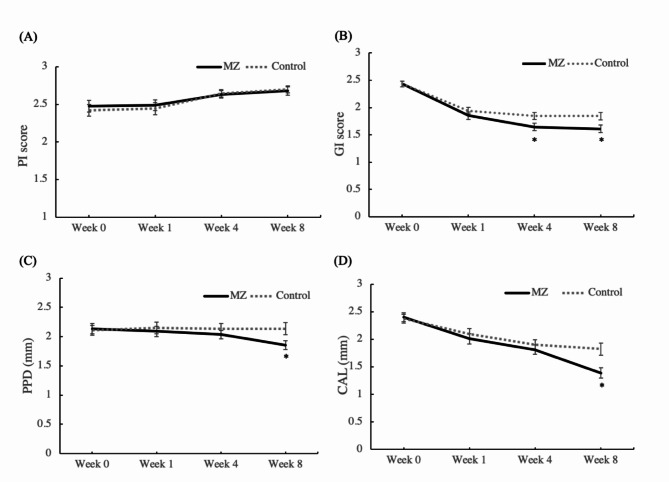
Comparison of clinical parameters within the same week. **(A)** Plaque index, **(B)** gingival index, **(C)** periodontal pocket depth, **(D)** clinical attachment loss. *significant difference compared to the control group at the same week (*p* < 0.05)

Intergroup comparisons of the PI, GI, PPD, and CAL at the same time points (Fig. 1) showed that the PI of the MZ group was not significantly different from that of the control group at any examination point. The GI of the MZ group was significantly better than that of the control group at 4 and 8 weeks PM (*p* < 0.05). The PPD and CAL at 8 weeks PM in the MZ group were significantly lower than the corresponding values in the control group (*p* < 0.05). Intergroup comparisons of BoP showed that the incidence of bleeding in the MZ group was significantly lower than that in the control group at 4 weeks PM (*p* < 0.05, Table 4).

**Table 4:** Comparison of the incidence of bleeding on probing (BoP) in the same week

Week	Group	BoP		Total	𝜒^2^ (*p*)
		Absent	Present		
Week 0	MZ	17 (18.9)	73 (81.1)	90 (100.0)	0.000 (1.000)
	Control	17 (18.9)	73 (81.1)	90 (100.0)	
Week 1	MZ	28 (31.1)	62 (68.9)	90 (100.0)	0.684 (0.408)
	Control	23 (25.6)	67 (74.4)	90 (100.0)	
Week 4	MZ	33 (36.7)	57 (63.3)	90 (100.0)	4.519^*^ (0.034)
	Control	20 (22.2)	70 (77.8)	90 (100.0)	
Week 8	MZ	33 (36.7)	57 (63.3)	90 (100.0)	0.620 (0.431)
	Control	28 (31.1)	62 (68.9)	90 (100.0)	

^*^p < 0.05

### Flow cytometric analysis and tissue cytokine expression

**Fig. 2:**
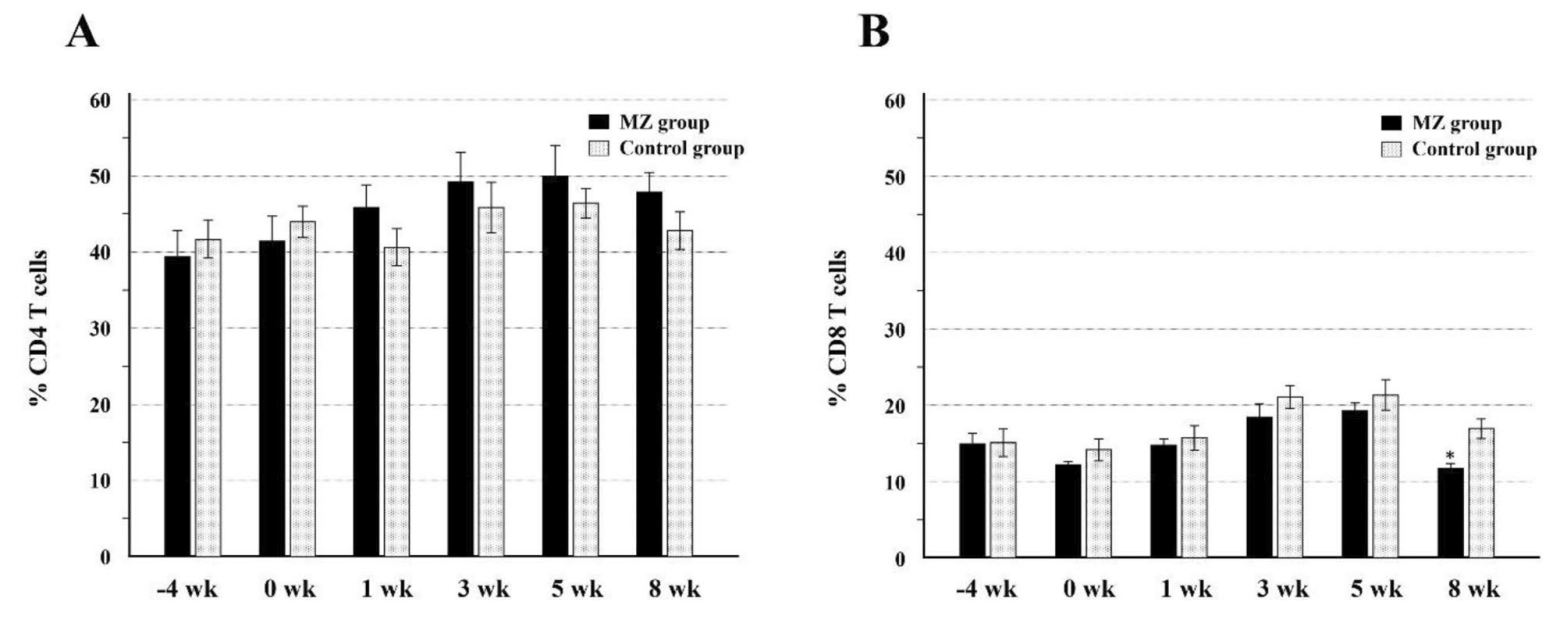
Changes in CD4 and CD8 T cell populations during the medication period. The data are presented as the mean percentage (with the standard error) of CD4 **(A)** or CD8 **(B)** T cells in the total PBMC population. *significant difference compared to the value of the control group at the same week (*p* < 0.05). PBMC, peripheral blood mononuclear cell

To evaluate the systemic effect of Magnoliae cortex and *Zea mays* L. extracts on the immune response, the percentages of CD4 + and CD8 + T cell populations in the total PBMCs were screened before (-4 and 0 weeks) and after (1, 3, 5, and 8 weeks) administration (Fig. 2). Throughout the medication period, the CD4 + T cell population increased, whereas the CD8 + T cell population decreased in the MZ group in comparison with the corresponding populations in the control group. However, the difference was not significant at any time point, except for the CD8 + T cell population at 8 weeks PM (*p* < 0.05, Fig. 2). The percentage of activated CD4 + or CD8 + T cells in the total CD4 + or CD8 + T cell population after treatment did not differ between the MZ and control groups (data not shown).

**Fig. 3:**
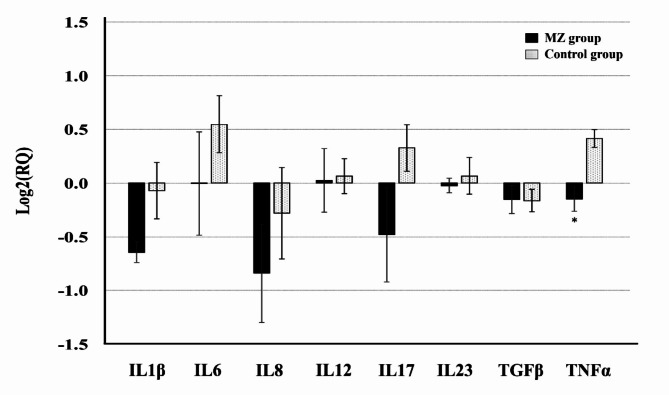
Comparison of cytokine transcription in gum tissue. The relative transcription at 8 wk PM was calculated using the value at 0 wk PM as the calibrator with a housekeeping gene (glyceraldehyde 3-phosphate dehydrogenase, GAPDH). Data are presented as the mean value of each group (error bars = standard deviation) at 8 wk PM compared to the baseline (0 wk). *significant difference compared to the value of the control group (*p* < 0.05). RQ, relative quantification; PM, post-medication

Transcriptional changes in cytokines in the inflamed tissue after treatment were measured and compared between the MZ and control groups (Fig. 3). Among the tested cytokines, interferon (IFN)-γ and IL-4 RNA were not detected by qRT-PCR (data not shown) and were excluded from presentation. In the MZ group, the expression of pro-inflammatory or inflammatory cytokines (IL-1β, IL-6, IL-12, IL-17, IL-23, and TNF-α) and an inflammatory chemokine (IL-8) was slightly downregulated after 8 weeks PM in comparison with the levels measured before medication. In contrast, these cytokine transcriptions were upregulated (IL-6, IL-17, and TNF-α) or remained unchanged in the control group at 8 weeks after ligature removal. TNF-α transcription significantly differed between the two groups (Fig. 3).

## Discussion

Periodontal disease is a common problem in veterinary practice. The prevalence of periodontal disease in dogs has been reported to be between 9.3% and 18.2% on the basis of visual assessment. However, careful examinations of anesthetized dogs showed that the prevalence was much higher, ranging from 44 to 100% [12]. Nevertheless, effective long-term treatment approaches for periodontal disease are limited.

Two decades ago, the effects of Magnolia cortex and *Zea mays* L. extract mixtures (formulation ratio, 2:1) on periodontitis were tested using experimentally induced periodontitis in beagle dogs [11]. The rationale of that study was based on the synergistic effect of Magnoliae cortex extract, which showed antimicrobial activity against periodontal pathogens, and *Zea mays* L. extract in improving gingival cellular activity [9]. However, the anti-inflammatory effects of Magnoliae cortex and *Zea mays* L. extracts were later demonstrated by several in vitro assays that tested them singly or together [6–8, 10]. Thus, the effects of the Magnoliae cortex and *Zea mays* L. extract mixture on systemic and local immune responses were not considered in the previous dog experiment [11]. Moreover, the previous periodontal animal experiments did not intend to evaluate veterinary use of this mixture, and instead used the animals as animal models of human periodontitis to replace a human in vivo challenge. Therefore, the formulation used in the previous study was based on human body weight and focused only on improving the clinical indices of periodontitis. According to these study results, the dosage of commercial MZ complex, which is used as an adjuvant in the treatment of human periodontitis, is 105 to 210 mg three times a day. In this study, a dose reduction of more than five times (60 mg/dog/day) the mixture used in the previous beagle experiment was administered orally to dogs to evaluate the long-term effects of this agent on periodontal clinical parameters and immunity as a trial for veterinary use [11].

The CBC and serum chemistry parameter values of all dogs remained within the normal range throughout the experiment, with or without medication, except below-normal potassium levels in one dog prior to the induction of periodontitis. However, this was thought to be a minor electrolyte imbalance due to water withdrawal during anesthetic preparation, and all subsequent serum chemistry values in this dog remained within the normal range. *Zea mays* L. extract has shown no toxic effects up to 500 mg/kg/day for 4 weeks, and Magnoliae cortex extract has been shown to be safe up to a dose of 240 mg/kg/day for 90 days in rats [13–15]. The results of this study also showed that long-term administration of a formulation containing low doses of Magnoliae cortex and *Zea mays* L. extracts may not induce any notable negative effects in clinically healthy dogs. Taken together, the findings of previous studies and the current study suggest that the formulation of Magnoliae cortex and *Zea mays* L. extracts at dosages used in this study could be administered to healthy dogs without any clinical side effects.

The baseline values of all clinical parameters were not significantly different between the groups. The GI values of both groups were significantly lower at 1 week in comparison with those at 0 weeks, and significant intergroup differences were observed at 4 and 8 weeks PM. The PPD and BoP of the MZ group significantly improved at 8 and 4 weeks PM, respectively, whereas those of the control group did not differ significantly throughout the experimental period. In addition, CAL gradually improved with time in both groups after ligature removal, and the MZ group showed a significant decrease at 8 weeks PM in comparison with the control group, while the PI in both groups increased over the examination period without showing a significant intergroup difference. Thus, even at doses much lower than those reported previously, the mixture of Magnoliae cortex and *Zea mays* L. extracts was associated with clinical improvement in periodontitis. Although not performed in this study, imaging or histological examinations could provide more confirmative evidence of the effect of mixture of Magnoliae cortex and *Zea mays* L. extracts on periodontitis in dogs. Further studies are needed to identify the regeneration of normal periodontal tissue.

Periodontal diseases are caused by host inflammatory responses against periodontal bacterial pathogens [1]. In recent studies, the anti-inflammatory effects of Magnoliae cortex and *Zea mays* L. extracts were demonstrated in single or combined tests in vitro [6–8, 10]. However, the effects of this mixture on systemic and local immune responses have not yet been evaluated in vivo. In this study, we monitored the frequencies of CD4 + and CD8 + T cells in both groups before and after treatment (Fig. 2). Throughout the experimental period, the two groups showed similar trends of frequency changes in CD4 + and CD8 + T cells in PBMCs. While the percentage of CD4 + T cells in the MZ group was higher than that in the control group after medication, the difference was not significant. In contrast, the percentage of CD8 + T cells was significantly lower than that in the control group at 8 weeks PM. In a human study, the frequency of CD4 + and CD8 + T cells in PBMCs was not related to the periodontitis status [16]. In a canine study, although the frequency of CD4 + and CD8 + T cells in the circulating blood of dogs with periodontitis was higher than that in healthy controls, the difference was not significant. Therefore, the findings did not clarify whether the decrease in the number of CD8 + T cells in the MZ group is clinically relevant for the treatment of periodontitis. Further studies are needed to elucidate the effect of Magnoliae cortex and *Zea mays* L. extracts on major immune cell subsets in the peripheral blood in periodontal diseases.

The balance of the local cytokine network is an important indicator of periodontitis in gingival tissues. However, the exact immunopathological mechanisms underlying periodontitis remain unclear. The general concept of Th1- and Th2-type CD4 + T cells established in other chronic inflammatory diseases is not suitable for periodontitis due to the contradictory results of previous studies [17]. However, the role of pro-inflammatory cytokines (IL-1, IL-6, and TNF-α) secreted by gingival tissue cells in response to pathogenic bacteria is relatively well-established [18, 19]. In this study, the local cytokine gene expression at 8 weeks PM was measured by qRT-PCR, and the changes in this value were calculated by comparing it with the value measured at 0 weeks, which was the time point of release of the ligatures used for inducing periodontitis. In the MZ group, the levels of IL-1β and TNF-α were slightly reduced while the IL-6 level did not change. In contrast, the levels of these pro-inflammatory cytokines were slightly elevated in the control group. However, the differences between the levels in the two groups was significant only for TNF-α. Moreover, administration of the Magnoliae cortex and *Zea mays* L. extract did not affect the expression of the Th1-type cytokines IL-12 and IFN-γ (not detected in this study, data not shown), the Th2-type cytokine IL-4 (not detected in this study, data not shown), and the regulatory cytokine transforming growth factor-β in comparison with the corresponding expression levels in the control group (Fig. 3). Among Th17-type cytokines, IL-17 expression in the control group was approximately 1.75 times that in the MZ group; however, the difference was not significant (Fig. 3). The inhibitory effect of MA on IL-1β, IL-16, and TNF-α secretion has been previously reported [10, 20]. However, inhibition of these pro-inflammatory cytokines was not clearly observed in this study. In addition, no changes in the levels of cytokines related to adaptive immune responses were observed. These findings can be explained by the animal model used in this study. The major immunopathological mechanism of periodontitis is the inflammatory host immune response to pathogenic microorganisms such as *Porphyromonas gingivalis*, *Treponema denticola*, and *Tannerella forsythia* [21, 22]. However, in this study, periodontitis was induced only by mechanical stimulation, and the device was removed at the time of medication. This may have limited the involvement of pathogenic adaptive host immune responses in the clinical progression of periodontitis. Consistent with this assumption, the transcription levels of the Th1 and Th2 cytokines IFN-γ and IL-4, respectively, were under the limit of detection by qRT-PCR even in the control group. Nevertheless, all pro-inflammatory cytokines showed a trend of reduced expression in comparison with their expression levels in the control group (Fig. 3). These results indicate that the in vivo anti-inflammatory effect of Magnoliae cortex and *Zea mays* L. extracts can reduce or inhibit the inflammatory immune response in periodontitis. This may have resulted in the improvement in clinical indicators in this study.

A limitation of this study is that the experimental model of periodontitis was only induced by mechanical stimulation in the absence of the causative pathogens shown in natural cases. Further studies are needed to test the effect in clinical cases of dog patients.

## Conclusion

Our findings indicate that the combination of Magnoliae cortex and *Zea mays* L. extracts may have a beneficial effect in the management of periodontal disease in dogs. Considering the problem of antimicrobial resistance, this formulation can serve as a novel supplement for long-term administration in periodontal dogs. Further studies are needed to examine the long-term effects of this combination in a clinical veterinary setting. In addition, a larger number of dogs should be included to provide more reliable statistics.

## Methods

### Ethical approval and experimental treatment

All animal experiments and study protocols were approved by the Institutional Animal Care and Use Committee of Knotus Co., Ltd. (Guri, Korea; approval no. KNOTUS IACUC 16-KE-121). Ten approximately 1-year-old healthy male beagles without periodontal disease were obtained from Xi’an Dilepu Biology & Medicine Co., Ltd. (Lianhu, China). None of the beagles were administered any systemic medications before starting the experiments. All experimental procedures, except jugular vein blood collection, were performed under general anesthesia with a combination of medetomidine (0.01 mg/kg; Orion Pharma, Finland), tramadol (2 mg/kg; Aju Pharm, Korea), and a commercial combination of zolazepam and tiletamine (2.5 mg/kg; Virbac, France) administered by intramuscular injection.

### Experimental induction of periodontitis and animal grouping

To prepare a healthy periodontium, all the teeth of the beagles were scaled and polished with a piezoelectric scaler (ART-SP2; Bonart Co., Ltd., Taiwan). For the next 2 weeks, a hard-pellet-type diet (Lab Animal Diet; Cargill Agri Purina Inc., Korea) was provided to the beagles to reduce the potential for plaque formation, and tooth brushing was performed after every meal to maintain a healthy periodontal status [23].

After the prophylactic period, experimental periodontitis was bilaterally induced using a twisted wire (Class One Orthodontics, USA) with a 2 − 0 silk (Ailee, Korea) ligature on the maxillary second, third, and fourth premolars as previously described [24]. To promote dental plaque formation, soft-moistened canned food (Cesar; Mars Inc., USA) was provided and tooth brushing was stopped for the following 8 weeks. The maintenance of the ligatures was checked daily, and unfastened ligatures were repaired immediately.

Magnolia cortex extract was purchased from Dongbang FTL (Seoul, Korea). Magnolia obovata thunberg bark was crushed with a blender, then 1 kg was taken and extracted twice for 3 h in a water bath at 65 °C using 5 L of 75% ethanol, cooled, and filtered. The two filtrates were combined and concentrated under reduced pressure to obtain 200 g of magnolol extract (not less than 0.5%, w/w). The *Zea mays* L. extract was prepared and manufactured by Dongkook Pharmaceutical Co., Ltd. (Seoul, Korea). Twelve kilograms of corn oil collected from corn kernels placed in a round flask, 52 L of ethanol, sodium hydroxide, and potassium hydroxide were added, and the mixture was stirred at 80 °C for 3 h and then cooled. The filtrate was concentrated, and 1.2 kg of blackish brown extract was obtained using ethyl acetate at room temperature, concentrated, and cooled to room temperature. It was filtered with ethanol and then concentrated again to obtain the titrated, unsaponifiable extract. The complex of Magnoliae cortex and *Zea mays* L. extracts were manufactured and provided by Dongkook Pharmaceutical Co., Ltd. (Seoul, Korea) in the form of capsules containing 40 mg and 20 mg of the two extracts, respectively.

After 8 weeks of periodontitis induction, the twisted ligatures were removed. The dogs were randomly divided into two groups of five dogs (30 teeth) each, and a researcher (SY Hwang) who did not participate in the clinical index evaluation confirmed that there was no significant difference in the baseline periodontal index of the two groups. One group was administered capsules containing the two extracts once daily for 8 weeks, whereas the other group received empty gelatin capsules (control group). Both groups were fed a hard-pellet diet until the end of the experiment.

### Sample collection and clinical evaluation

Ten milliliters of blood were collected from the jugular vein at 4 and 8 weeks post-periodontitis induction and 1, 3, 5, and 8 weeks post-medication (PM) to evaluate the complete blood count (CBC) and serum chemistry values and isolate peripheral blood mononuclear cells (PBMCs). The following clinical periodontal parameters were assessed under general anesthesia at baseline (week 0) and at 1, 4, and 8 weeks PM: plaque index (PI; range, 0–3), gingival index (GI; range, 0–3), periodontal pocket depth (PPD; mm, normal range: less than 3 mm), clinical attachment loss (CAL; mm), and bleeding on probing (BoP; score range, 0–1) [5, 25, 26]. The parameters were measured at four sites per tooth (meiobuccal, mid buccal, mid palatal, and mesiobuccal sites) using a manual periodontal probe. For tissue cytokine expression, a 5 mm × 5 mm gingival sample was obtained from the buccal gingival margins of the right maxillary first molar and right maxillary fourth premolar level of each dog at 0 and 8 weeks PM, respectively. The obtained samples were washed immediately with 0.9% NaCl to remove debris and blood and snap-frozen in liquid nitrogen. The tissue samples were stored at -70 °C until processing.

All dental experimental procedures were performed by a single experienced clinician (SE Kim) using a Williams periodontal probe (XP23-W Williams Explorer-Probe; Osung, Korea).

### Flow cytometric analysis

Blood samples were collected from each animal in heparinized vacuum containers at the time points described above. PBMCs were isolated from the samples using a mixture of Ficoll-Hypaque media at densities of 1.119 g/mL and 1.077 g/mL (Sigma Chemical Co., USA), as described previously [27]. The isolated PBMCs were labeled with rat anti-canine CD4 monoclonal antibody conjugated with fluorescein isothiocyanate (catalog No. 11-5040-42; eBiosciences, Frankfurt am Main, Germany) or rat anti-canine CD8 monoclonal antibody conjugated with PerCP-eFluor™ 710 (catalog No. 46-5080-42; eBiosciences, Frankfurt am Main, Germany) in combination with rat anti-canine CD25 monoclonal antibody conjugated with phycoerythrin (catalog No. 12-0250-42; eBiosciences, Frankfurt am Main, Germany). The percentages of activated CD4 + and CD8 + T cells in the total CD4 + and CD8 + T cell pools were measured using an electronic gating strategy to isolate the CD4 and CD8 populations for analysis [28]. Briefly, 10^6^ canine PBMCs were distributed in a 96-well conical bottom assay plate with 5 µL of each monoclonal antibody in a final volume of 200 µL of phosphate-buffered saline containing 20% acid-citrate-dextrose, 0.5% horse serum, and 0.09% azide. After incubation for 15 min, the PBMCs were washed three times with phosphate-buffered saline containing 20% acid-citrate-dextrose and fixed in phosphate-buffered saline containing 2% formaldehyde. The fixed cells were stored at 4℃ until processing. The stained cells were evaluated by flow cytometry using a FACSCalibur instrument (Becton Dickinson, USA), and CellQuest software (Becton Dickinson, USA) was used to collect the data. FCS Express software ver. 6 (De Novo Software, Thornton, Ontario, Canada) was used to analyze the data [29].

### Quantitative real-time polymerase chain reaction for comparison of tissue cytokine expression

The frozen gingival tissue sample was thawed in a 2-mL screw-cap tube containing 1 mL of cooled TRIzol^®^ reagent (Life Technologies, CA) and 0.1-mm zirconia-silica beads (BioSpec Products, Inc., OK). Mechanical disruption was performed using a FastPrep-24 instrument at a speed setting of 6.5 for 45 s. After centrifugation at 12,000 × *g* for 10 min, the supernatant was collected, and total RNA was extracted using Direct-zol™ RNA miniprep (Zymo Research, CA). The extracted RNA was treated with a Turbo™ DNase kit (Thermo Fisher Scientific, Lithuania) to remove residual DNA contamination. cDNA was generated by reverse transcription using a High-Capacity cDNA Reverse Transcription kit (Thermo Fisher Scientific, Lithuania) according to the manufacturer’s recommendations.

Quantitative real-time polymerase chain reaction (qRT-PCR) was performed on a StepOnePlus Real-Time PCR System (Applied Biosystems, CA, USA) using Power SYBR Green PCR Master Mix (Applied Biosystems). The primer set used in this study is listed in Table 5 [30, 31]. The qRT-PCR conditions and data collection method have been described previously [32]. The relative quantification (RQ) values of gene transcription in gum tissue were compared using the 2^−(∆∆*Ct*)^ method [33]. Briefly, the ∆*Ct* values of each cytokine from samples collected at 8 weeks PM were calculated in comparison with the mean *Ct* value of the housekeeping gene glyceraldehyde 3-phosphate dehydrogenase (GAPDH). Then, the ∆∆*Ct* values were calculated in comparison with the mean ∆*Ct* values of samples collected from the same group at 0 weeks PM, and these values served as the calibrators. The results are expressed as the RQ values of transcription in comparison with those in the control group.

**Table 5:** Primers used for qRT-PCR quantification for cytokine transcription

Gene	Forward primer	Reverse primer
GAPDH	ATCACTGCCACCCAGAAGAC	TCAGCTCAGGGATGACCTTG
IL-1β	CAAGTCTCCCACCAGCTCTGTA	GGGCTTCTTCAGCTTCTCCAA
IL-4	TCACCAGCACCTTTGTCCAC	CGCTTGTGTTCTTTGGAGCA
IL-6	TTAAGTACATCCTCGGCAAAATCT	CAGTGCCTCTTTGCTGTCTTCA
IL-8	CTCTCTGTGAAGCTGCAGTTCTG	T GGAAAGGTGTGGAGTGTGTTTTT
IL-12	GTGCCTCAACCACTCCCAA	CAATCTCTTCGGAAGTGCAGG
IL-17	GGAATCTGCACCGCAATGAGGAC	CGCAGAACCAGGATCTCTTGCTGG
IL-23	CAAGGGGAGAAAAACAGCAG	TGCTGTCCGTTCTGTGAGTC
IFN-γ	GCGCAAGGCGATAAATGAAC	CTGACTCCTTTTCCGCTTCC
TGF-β	CTGGAGTCGTGAGGCAGTG	GCAGTGTGTTATCTTTGCTGTCA
TNF-α	TCTCGAACCCCAAGTGACAAG	CAACCCATCTGACGGCACTA

qRT-PCR, quantitative real-time polymerase chain reaction; GAPDH, glyceraldehyde 3-phosphate dehydrogenase; IL, interleukin; TNF, tumor necrosis factor.

### Statistical analysis

All data were statistically analyzed using a commercial software program (SPSS 26.0; SPSS, USA). To evaluate the differences in clinical parameters at different time points, the corresponding values were assessed using repeated-measures analysis of variance. To compare the periodontal status between the groups, the PI, GI, PPD, and CAL values at each time point were compared using the Student’s *t*-test, and the BoP values were compared using the Chi-square test. The results of the flow cytometric analysis (percentage of CD4 + and CD8 + T cells) and the RQ values of gene transcription were analyzed using one-way analysis of variance. In all tests, a *p* value less than 0.05 was considered significant.

## Electronic supplementary material

Below is the link to the electronic supplementary material.

Supplementary Material 1. Figure S1. Photographs of gum tissues after medication. The photographs of right and left side-gums of individual dogs in control (A) and MZ (B) groups were taken at 0, 1, 4, and 8 wk post-medication.

Supplementary Material 2

## Data Availability

The data presented in this study are available on request from the corresponding author.

## Abbreviations

BoP: Bleeding on probing
CAL: Clinical attachment loss
CBC: Complete blood count
GAPDH: Glyceraldehyde 3-phosphate dehydrogenase
GI: Gingival index
IFN: Interferon
IL: Interleukin
PBMC: Peripheral blood mononuclear cell
PI: Plaque index
PM: Post-medication
PPD: Periodontal pocket depth
qRT-PCR: Quantitative real-time polymerase chain reaction
RQ: Relative quantification
TNF: Tumor necrosis factor
