# Supplementary figures and images for: The anti-inflammatory effect of a magnoliae cortex and Zea mays L. extract mixture in a canine model of ligature-induced periodontitis
Source: BMC Vet Res. 2024 Sep 28;20:437. doi: 10.1186/s12917-024-04243-0 (PMC11437871; doi:10.1186/s12917-024-04243-0)

Fig. S1A 0 wk 1 wk PM 4 wk PM 8 wk PM


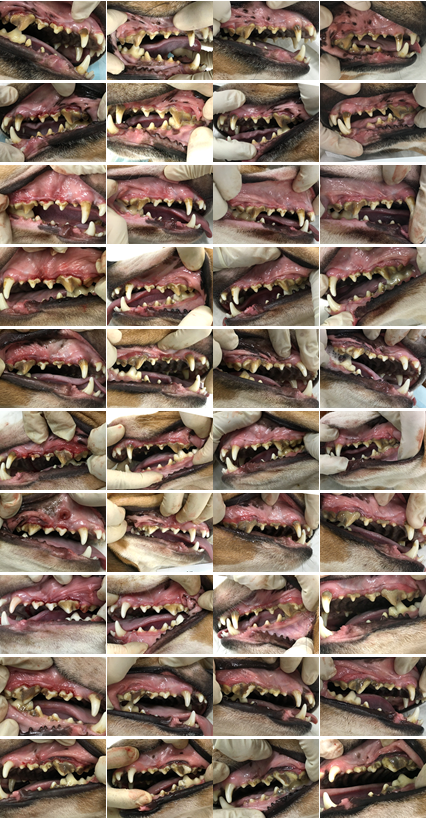


C 1-R

C 1-L

C 2-R

C 2-L

C 3-R

C 3-L

C 4-R

C 4-L

C 5-R

C 5-L

Supplement: Supplementary file 1 — Supplementary Material 1. Figure S1. Photographs of gum tissues after medication. The photographs of right and left side-gums of individual dogs in control (A) and MZ (B) groups were taken at 0, 1, 4, and 8 wk post-medication. [file 12917_2024_4243_MOESM1_ESM.docx]

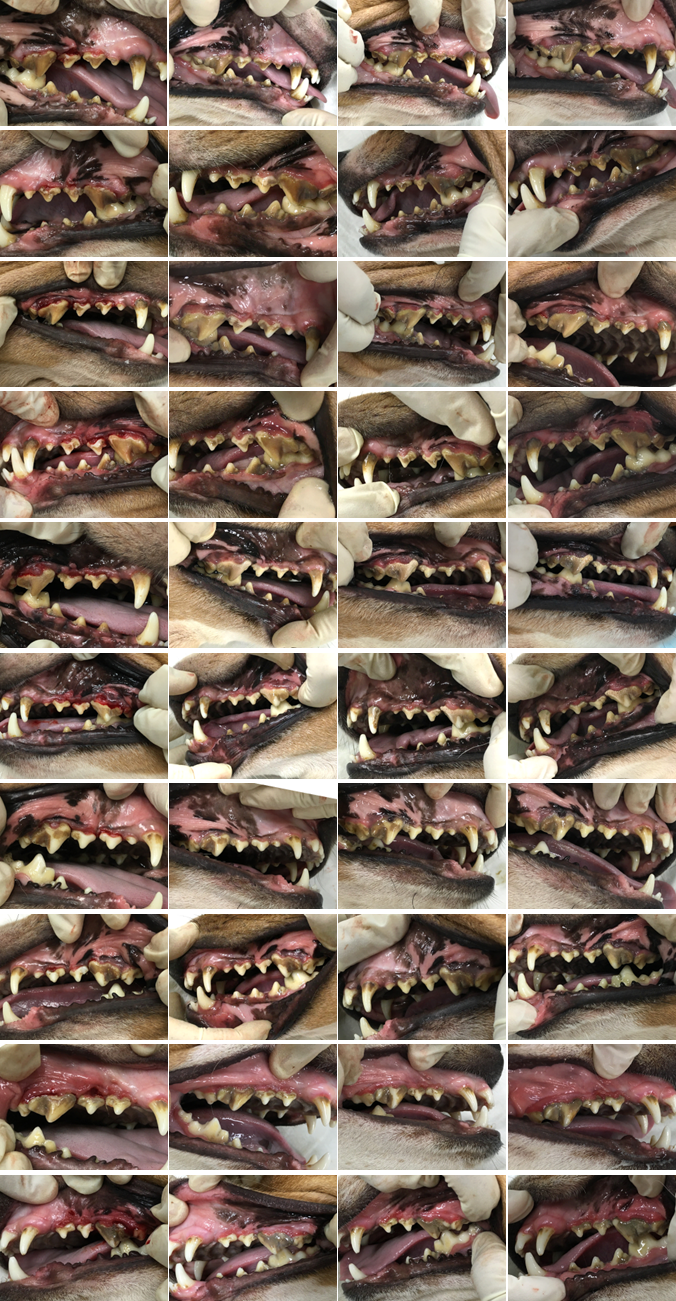
Fig. S1B 0 wk 1 wk PM 4 wk PM 8 wk PM

MZ 1-R

MZ 1-L

MZ 2-R

MZ 2-L

MZ 3-R

MZ 3-L

MZ 4-R

MZ 4-L

MZ 5-R

MZ 5-L

Supplement: Supplementary file 2 — Supplementary Material 2 [file 12917_2024_4243_MOESM2_ESM.docx]
